# Insights into phenotypic differences between humans and mice with p.T721M and other C-terminal variants of the *SLC26A4* gene

**DOI:** 10.1038/s41598-021-00448-7

**Published:** 2021-10-25

**Authors:** Chin-Ju Hu, Ying-Chang Lu, Cheng-Yu Tsai, Yen-Hui Chan, Pei-Hsuan Lin, Yi-Shan Lee, I.-Shing Yu, Shu-Wha Lin, Tien-Chen Liu, Chuan-Jen Hsu, Ting-Hua Yang, Yen-Fu Cheng, Chen-Chi Wu

**Affiliations:** 1 Department of Otolaryngology, National Taiwan University Hospital, 7 Chung-Shan S. Rd., Taipei, 100 Taiwan; 2 Program in Speech and Hearing Bioscience and Technology, Harvard Medical School, Boston, MA 02115 USA; 3 Department of Otolaryngology, Taichung Tzu Chi Hospital, Buddhist Tzu Chi Medical Foundation, Taichung, 427 Taiwan; 4 Transgenic Mouse Models Core (TMMC), Division of Genomic Medicine, Research Center for Medical Excellence, National Taiwan University, Taipei, 100 Taiwan; 5 Department of Otolaryngology, College of Medicine, National Taiwan University, Taipei, 100 Taiwan; 6 Department of Medical Research, Taipei Veteran General Hospital, 201, Sec.2, Shi-Pai Rd, Taipei, 112 Taiwan; 7 Department of Otolaryngology-Head and Neck Surgery, Taipei Veteran General Hospital, Taipei, 112 Taiwan; 8 School of Medicine, National Yang Ming Chiao Tung University, Taipei, 112 Taiwan; 9 Department of Medical Research, National Taiwan University Hospital Hsin-Chu Branch, Hsinchu, 302 Taiwan

**Keywords:** Animal biotechnology, Genomics, Protein sequencing, Genetics research

## Abstract

Recessive variants of the *SLC26A4* gene are an important cause of hereditary hearing impairment. Several transgenic mice with different *Slc26a4* variants have been generated. However, none have recapitulated the auditory phenotypes in humans. Of the *SLC26A4* variants identified thus far, the p.T721M variant is of interest, as it appears to confer a more severe pathogenicity than most of the other missense variants, but milder pathogenicity than non-sense and frameshift variants. Using a genotype-driven approach, we established a knock-in mouse model homozygous for p.T721M. To verify the pathogenicity of p.T721M, we generated mice with compound heterozygous variants by intercrossing *Slc26a4*^+*/T721M*^ mice with *Slc26a4*^*919-2A*>*G/919-2A*>*G*^ mice, which segregated the c.919-2A > G variant with abolished *Slc26a4* function. We then performed serial audiological assessments, vestibular evaluations, and inner ear morphological studies. Surprisingly, both *Slc26a4*^*T721M/T721M*^ and *Slc26a4*^*919-2A*>*G/T721M*^ showed normal audiovestibular functions and inner ear morphology, indicating that p.T721M is non-pathogenic in mice and a single p.T721M allele is sufficient to maintain normal inner ear physiology. The evidence together with previous reports on mouse models with *Slc26a4* p.C565Y and p.H723R variants, support our speculation that the absence of audiovestibular phenotypes in these mouse models could be attributed to different protein structures at the C-terminus of human and mouse pendrin.

## Introduction

Recessive variants in the *SLC26A4* (PDS, GeneID: 5172) gene are a common cause of hereditary hearing impairment (HHI)^1^. In certain populations, *SLC26A4* variants can be identified in approximately 15% to 20% of patients with HHI^2^. *SLC26A4* encodes pendrin, a chloride/iodide/bicarbonate transporter expressed in the thyroid, inner ears, kidneys, lungs, liver, and heart^3,4^. Recessive *SLC26A4* variants lead to Pendred syndrome (PS; MIM #274,600) and non-syndromic DFNB4 (MIM# 600,791). DFNB4 is characterized by isolated sensorineural hearing impairment (SNHI), which is associated with a common inner ear malformation called enlarged vestibular aqueduct (EVA; MIM 603,545), whereas patients with PS have goiter in addition to EVA^5^. To date, approximately 700 *SLC26A4* variants have been identified (https://databases.lovd.nl/shared/genes/SLC26A4). Clinically, patients with *SLC26A4* variants, either with DFNB4 or PS, usually suffer from progressive or fluctuating SNHI^6^.

The pathogenesis of SNHI in patients with DFNB4 and PS has been partially elucidated in various mouse models. Several mouse models have been generated, including knock-out *Slc26a4*^*-/-*^ mice^7^, *Slc26a4*^*loop/loop*^ mice with the p.S408F variant^8^, *Slc26a4*^*919-2A*>*G9/919-2A*>*G*^ mice with the c.919–2 A > G variant^9^, *Slc26a4*^*H723R/H723R*^ mice with the p.H723R variant^10^, *Slc26a4*^*L236P/L236P*^ mice with the p.L236P variant^11^, *Slc26a4*^*C565Y/C565Y*^ mice with p.C565Y variant^12^, conditional knock-out Tg[E]; Tg[R]; *Slc26a4*^*Δ/Δ*^ mice^13^, and humanized hH723R Tg mice with the p.H723R variant in the human *SLC26A4* sequence^14^. None of these models recapitulated SNHI phenotypes in humans. Knock-out *Slc26a4*^*-/-*^, *Slc26a4*^*loop/loop*^, and *Slc26a4*^*919-2A*>*G9/919-2A*>*G*^ mice showed congenital profound SNHI that was too severe compared to their human counterparts^7–9^. *Slc26a4*^*H723R/H723R*^ and *Slc26a4*^*C565Y/C565Y*^ mice showed normal hearing without any hearing loss phenotypes^10,12^. Although Tg[E]; Tg[R]; *Slc26a4*^*Δ/Δ*^ mice demonstrated hearing loss of various severities, doxycycline was required to induce the phenotype^13^. The auditory phenotypes of *Slc26a4*^*L236P/L236P*^ and hH723R Tg mice were milder than those of *Slc26a4*^*-/-*^, *Slc26a4*^*loop/loop*^, and *Slc26a4*^*919-2A*>*G9/919-2A*>*G*^ mice^11,14^, yet the absence of progressive hearing loss could not reflect the clinical symptoms in patients with *SLC26A4* mutations.

**Figure 1:**
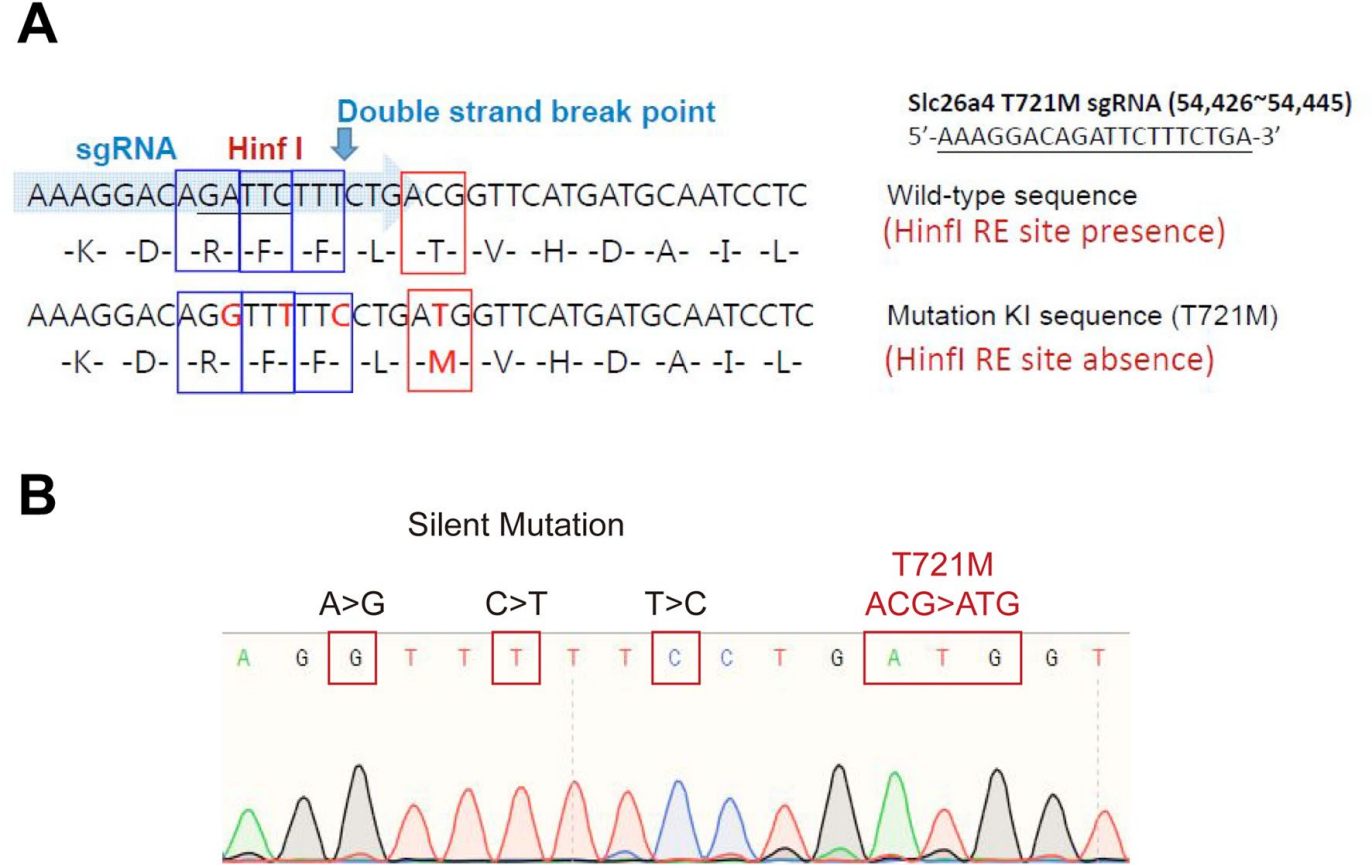
Generation of mice with *Slc26a4* p.H721M variant using CRISPR/Cas9. (**A**) Design diagram. SgRNA for CRISPR/Cas9 and silent mutations for enzyme cutting sites as a check were designed to incorporate the c.2162C > T (p.H721M) variant into the genome of C57BL/6 mice. To generate the *Slc26a4* p.T721M variant, the codon “ACG” was mutated to “ATG”. (**B**) Sanger sequencing was performed to confirm the nucleotide change in transgenic mice. The sequence was read in reverse.

Parallel to the transgenic mouse models, studies on cell lines have provided crucial insights into the pathogenicity of *SLC26A4* variants. It has been reported that different *SLC26A4* variants may result in different degrees of protein misexpression and/or dysfunction^15–17^. Among the *SLC26A4* variants, whose pathogenicity has been investigated in cell lines, the p.T721M variant is especially interesting. The expression of pendrin in cell lines with p.T721M could not be rescued after salicylate treatment, but that in cell lines with p.H723R could, indicating that p.T721M might confer a stronger pathogenicity than p.H723R^15^. Based on these results, we hypothesized that mice with the *Slc26a4* p.T721M variant might exhibit auditory phenotypes milder than those of c.919-2A > G mice but more severe than those of p.H723R mice. In this study, we generated a knock-in mouse model with the p.T721M variant, as well as compound heterozygous (*Slc26a4*^*919-2A*>*G/T721M*^) mice in which we tried to manipulate the severity of phenotypes by abolishing the other functional *Slc26a4* allele^9^. We then characterized the audiovestibular phenotypes and inner ear pathology in these mouse models (Fig. 1).

## Results

### Auditory phenotypes

**Figure 2:**
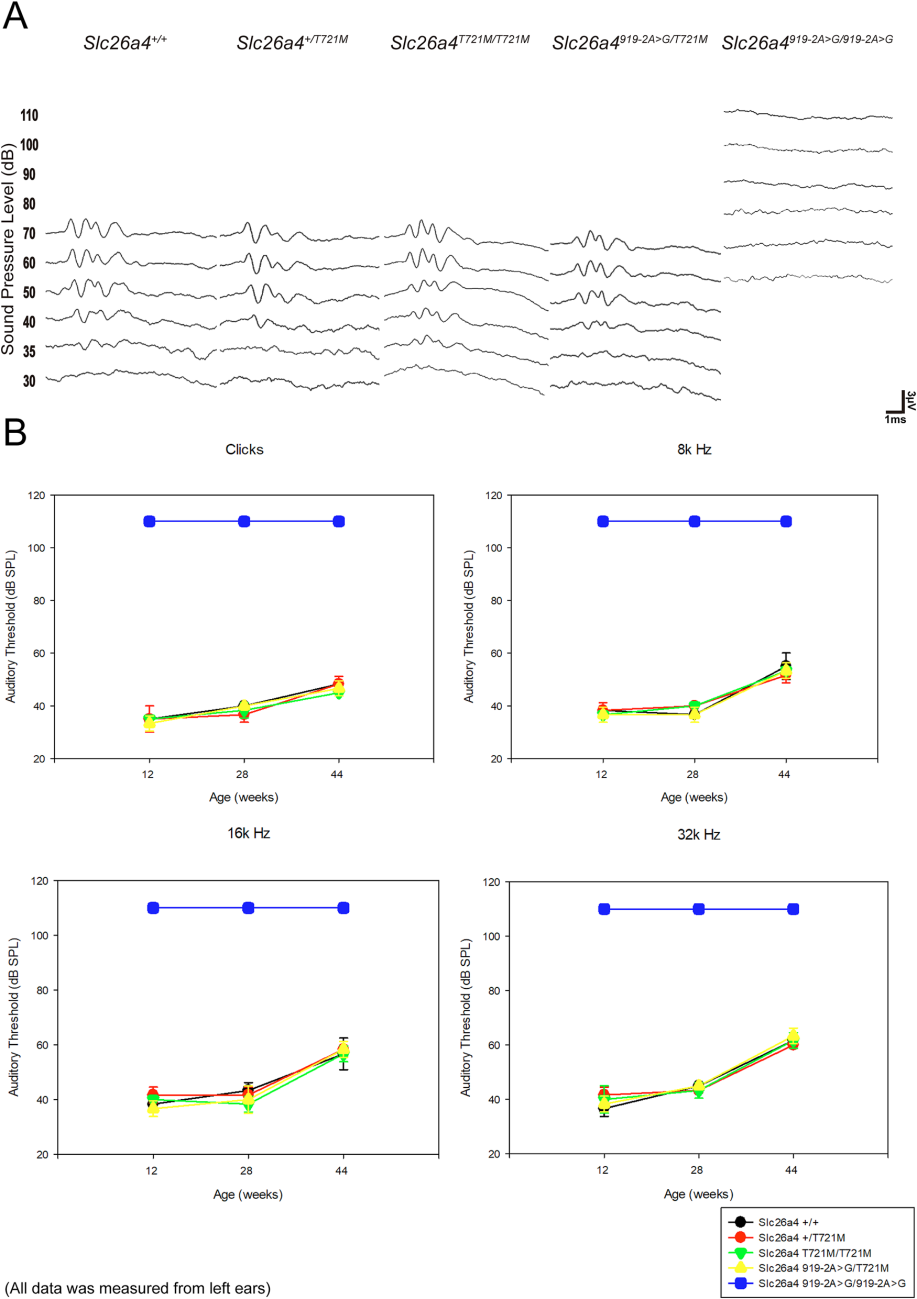
Auditory phenotypes. (**A**) The ABR waveforms in clicks did not differ significantly among heterozygous *Slc26a4*^+*/T721M*^, homozygous *Slc26a4*^*T721M/T721M*^, compound heterozygous *Slc26a4*^*919-2A*>*G/T721M*^, and wild-type *Slc26a4*^+*/*+^ mice. The ABR waveforms of *Slc26a4*^*919-2A*>*G/919-2A*>*G*^ mice with profound hearing loss were also determined for comparison. (**B**) Heterozygous, homozygous, and compound heterozygous mice showed normal hearing thresholds across different frequencies, similar to those in wild-type *Slc26a4*^+*/*+^ mice. There were no significant differences among these four groups of mice at 12, 28, and 44 weeks.

Wild-type mice (*Slc26a4*^+*/*+^), heterozygous mice (*Slc26a4*^+*/T721M*^), and homozygous mice (*Slc26a4*^*T721M/T721M*^) (n = 10 each) were subjected to audiological evaluations at 12, 28, and 44 weeks (Fig. 2). Both *Slc26a4*^+*/T721M*^ and *Slc26a4*^*T721M/T721M*^ mice had normal hearing up to 44 weeks, indicating that the p.T721M allele did not cause deafness in mice.

To confirm the pathogenicity of the p.T721M allele in mice, we invalidated the *Slc26a4* allele by intercrossing *Slc26a4*^+*/T721M*^ mice with *Slc26a4*^*919-2A*>*G/919-2A*>*G*^ mice to generate compound heterozygous mice (i.e., *Slc26a4*^*919-2A*>*G/T721M*^)^9^. Similar to heterozygous mice with the c.919-2A > G variant (i.e., *Slc26a4*^+*/919–2-2A*>*G*^), *Slc26a4*^*919-2A*>*G/T721M*^ mice (n = 10) had normal hearing up to 44 weeks. Furthermore, physical collisions such as falling from rotarods did not induce hearing loss in *Slc26a4*^*T721M/T721M*^ and *Slc26a4*^*919-2A*>*G/T721M*^ mice (data not shown). These findings indicate that the p.T721M allele is not pathogenic and a single allele with p.T721M is sufficient to maintain the auditory function in mice with the p.T721M variant.

### Inner ear morphology and pendrin expression

**Figure 3:**
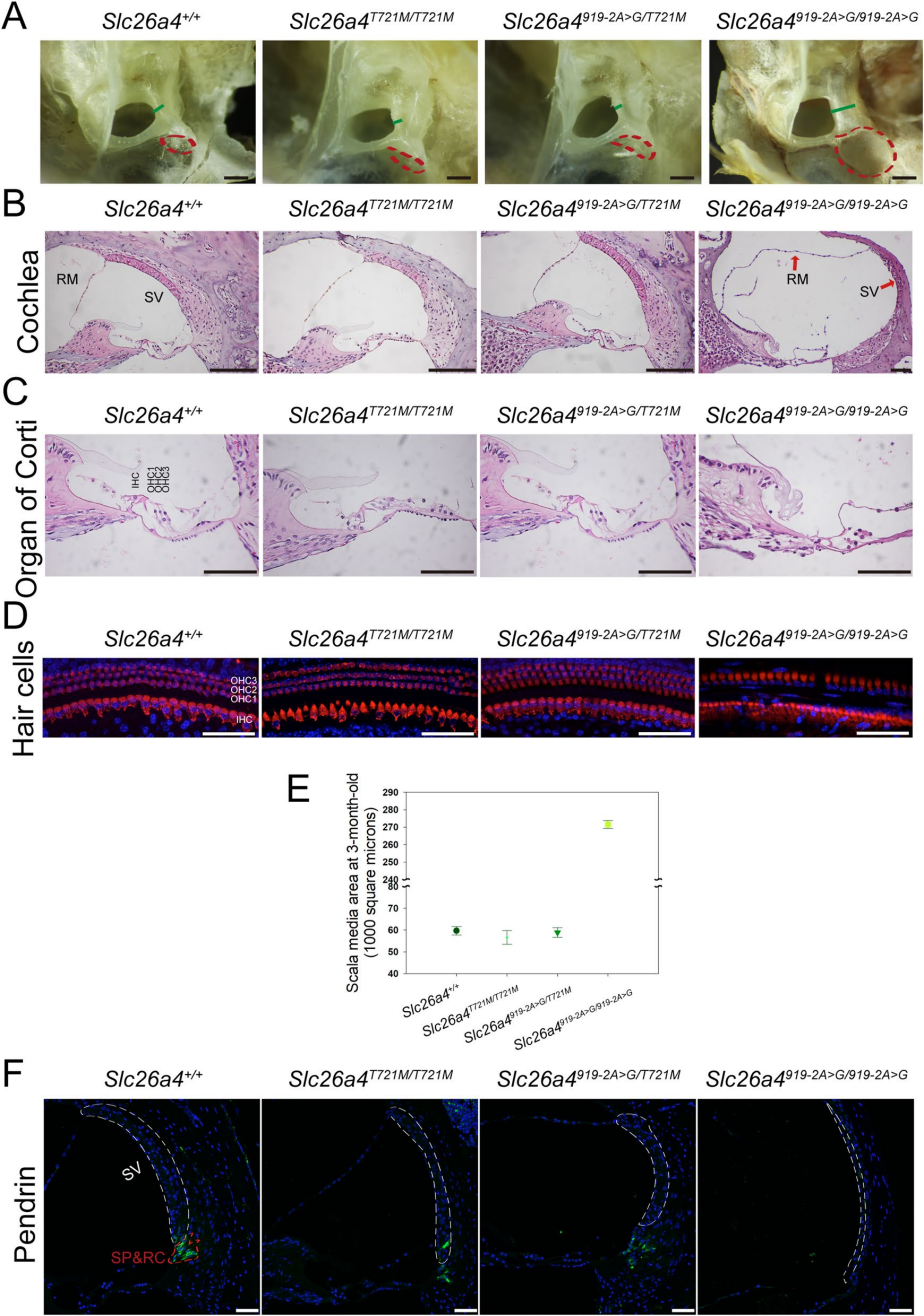
Cochlea histology. (**A**) Gross morphology of the vestibular aqueduct and endolymphatic sac. The vestibular aqueduct and endolymphatic sac were enlarged in *Slc26a4*^*919-2A*>*G/919-2A*>*G*^ mice. The length of the green lines represents the width of vestibular aqueduct. The red dash lines denote the contour of the sac. (**B**) Histology of the cochlea harvested from 3-month-old mice. On histological examination, the scala media was dilated in *Slc26a4*^*919-2A*>*G/919-2A*>*G*^ mice, but not in *Slc26a4*^*T721M/T721M*^ and *Slc26a4*^*919-2A*>*G/T721M*^ mice (RM, Reissner's membrane; SV, stria vascularis; bar = 150 μm). (**C**) In the view of the organ of Corti panel, degeneration of hair cells was observed in *Slc26a4*^*919-2A*>*G/919-2A*>*G*^ mice, but not in the other three groups of mice (IHC, inner hair cells; OHC 1–3, three rows of outer hair cells; bar = 150 μm). (**D**) Histology of the cochlear hair cells harvested from 3-month-old mice. Myosin VIIA expression was normal in *Slc26a4*^*T721M/T721M*^ and *Slc26a4*^*919-2A*>*G/T721M*^ mice, but was diminished in *Slc26a4*^*919-2A*>*G/919-2A*>*G*^ mice (red: Myosin VIIA, blue: DAPI; bar = 50 μm). (**E**) Quantitative analysis of endolymphatic space. The cross-sectional area of the scala media (middle turn) in *Slc26a4*^*919-2A*>*G/919-2A*>*G*^ mice was significantly larger than that in the other three groups. (**F**) The histology and expression of pendrin in the stria vascularis. Significant atrophy of stria vascularis and poor protein expression of pendrin were observed in *Slc26a4*^*919-2A*>*G/919-2A*>*G*^ mice. In contrast, pendrin was normally distributed in the spiral prominence (SP) and root cells (RC) in *Slc26a4C*^*T721M/T721M*^ and *Slc26a4*^*919-2A*>*G/T721M*^ mice, similar to that in wild-type mice. Tissues were harvested from 3-month-old mice (green: pendrin, white dash line: the contour of stria vascularis; bar = 50 μm).

Cochlear morphology was investigated in homozygous (*Slc26a4*^*T721M/T721M*^) and compound heterozygous mice (*Slc26a4*^*919-2A*>*G/T721M*^). Cochlear morphologies of wild-type mice and profoundly deaf *Slc26a4*^*919-2A*>*G/919-2A*>*G*^ mice were also obtained for comparison. The endolymphatic sac was severely enlarged only in *Slc26a4*^*919-2A*>*G/919-2A*>*G*^ mice, and remained normal in size in *Slc26a4*^*T721M/T721M*^ and *Slc26a4*^*919-2A*>*G/T721M*^ mice (Fig. 3A). Similarly, abnormal morphological findings in *Slc26a4*^*919-2A*>*G/919-2A*>*G*^ mice, including dilatation of the scala media, atrophy of the stria vascularis, and degeneration of the cochlear hair cells, were not observed in *Slc26a4*^*T721M/T721M*^ and *Slc26a4*^*919-2A*>*G/T721M*^ mice (Fig. 3B–D). Quantitative analyses of the endolymphatic space revealed that the cross-sectional area of the scala media in *Slc26a4*^*919-2A*>*G/919-2A*>*G*^ mice was significantly larger than those in wild-type, *Slc26a4*^*T721M/T721M*^, and *Slc26a4*^*919-2A*>*G/T721M*^ mice (271.6 ± 2.2; 59.6 ± 2.0; 56.5 ± 3.1; 58.9 ± 2.2 *1000 μm^2^, respectively, n = 3 each) (Fig. 3E).

We then examined the expression of pendrin in the cochlea of *Slc26a4*^*T721M/T721M*^ and *Slc26a4*^*919-2A*>*G/T721M*^ mice by immunolocalization (Fig. 3F). In both strains, pendrin was normally distributed in the spiral prominence and root cells, indicating that the expression of pendrin was normal in the p.T721M mice. In addition, the contour of the stria vascularis (indicated by white dashed lines in the figure) showed atrophic changes in *Slc26a4*^*919-2A*>*G/919-2A*>*G*^ mice, but not in *Slc26a4*^*T721M/T721M*^ and *Slc26a4*^*919-2A*>*G/T721M*^ mice.

### Vestibular function evaluation

**Figure 4:**
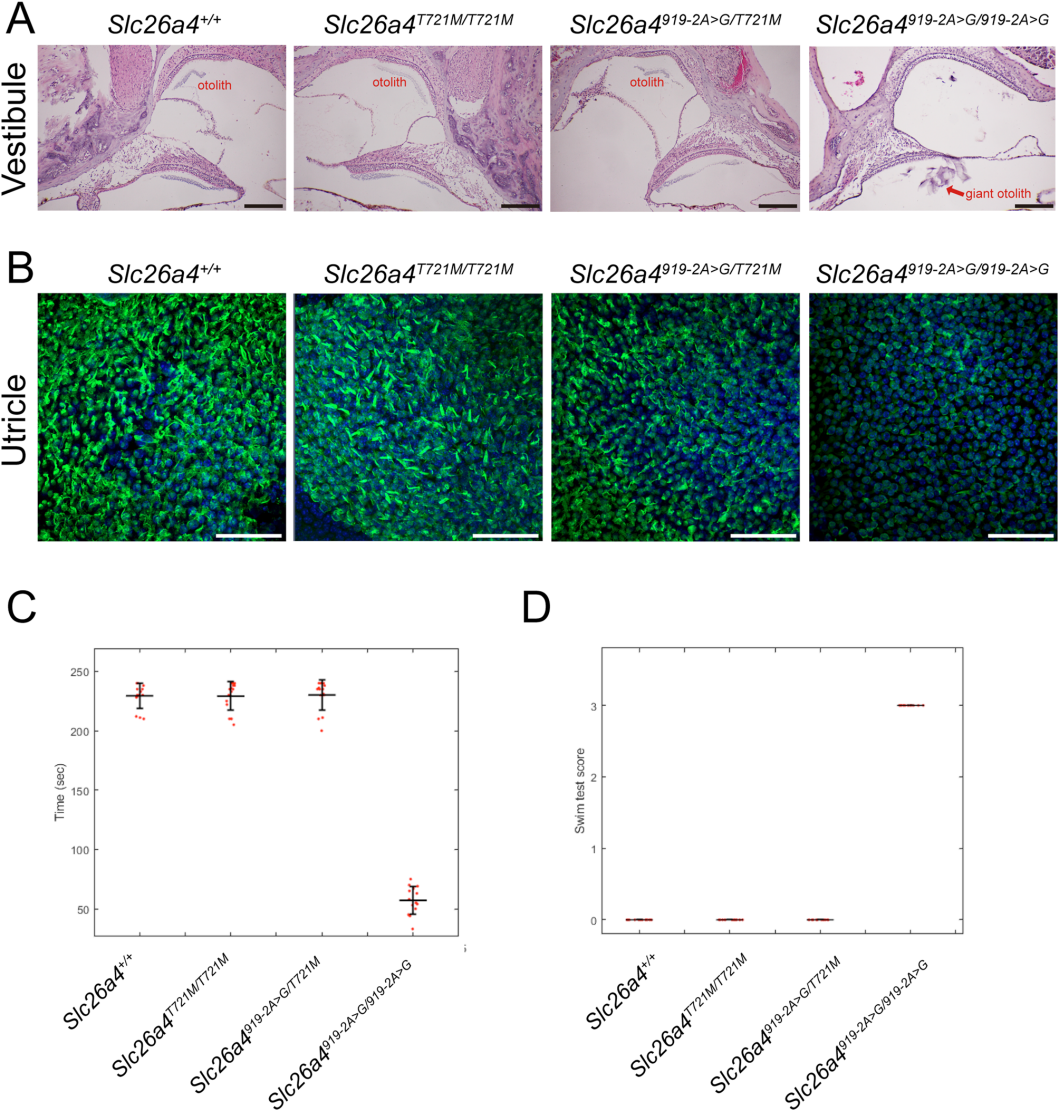
Morphology, histology, and phenotypes of the vestibule system. (**A**) Histology of the vestibular organs. Giant otoconia was observed in *Slc26a4*^*919-2A*>*G/919-2A*>*G*^ mice, but the otoconia was normal in *Slc26a4*^*T721M/T721M*^ and *Slc26a4*^*919-2A*>*G/T721M*^ mice (bar = 150 μm). (**B**) Fluorescence confocal microscopy observations. In contrast to the *Slc26a4*^*919-2A*>*G/919-2A*>*G*^ mice, vestibular hair cells in *Slc26a4*^*T721M/T721M*^ and *Slc26a4*^*919-2A*>*G/T721M*^ mice were not degenerated (green: Myosin VIIA; bar = 50 μm). (**C–D**) In contrast to the *Slc26a4*^*919-2A*>*G/919-2A*>*G*^ mice, the homozygous *Slc26a4*^*T721M/T721M*^ and compound heterozygous *Slc26a4*^*919-2A*>*G/T721M*^ mice performed well in rotarod test (**C**) and swimming test (**D**), similar to wild-type *Slc26a4*^+*/*+^ mice.

Vestibular morphology was also investigated in homozygous mice (*Slc26a4*^*T721M/T721M*^) and compound heterozygous mice (*Slc26a4*^*919-2A*>*G/T721M*^) (Fig. 4A). Both strains of mice showed normal vestibule morphologies. In contrast, giant otoliths were observed in *Slc26a4*^*919-2A*>*G/919-2A*>*G*^ mice. Fluorescence confocal microscopy demonstrated that vestibular hair cells in *Slc26a4*^*T721M/T721M*^ and *Slc26a4*^*919-2A*>*G/T721M*^ mice did not degenerate (Fig. 4B).

Fifteen mice of each group including *Slc26a4*^+*/*+^, *Slc26a4*^+*/T721M*^, *Slc26a4*^*T721M/T721M*^, and *Slc26a4*^*919-2A*>*G/T721M*^ mice, were subjected to vestibular evaluations. Similar to the normal audiological phenotypes, neither heterozygous mice (i.e., *Slc26a4*^+*/T721M*^ and *Slc26a4*^*919-2A*>*G/T721M*^) nor homozygous mice (i.e., *Slc26a4*^*T721M/T721M*^) showed vestibular deficits such as head tilting and circling behavior, and both groups performed normally on the rotarod (Fig. 4C) and in swimming tests (Fig. 4D). These findings indicate that a single p.T721M allele is sufficient to maintain normal vestibular function in mice.

## Discussion

The knock-in mouse generated in this study, the *Slc26a4*^*T721M/T721M*^ with the *Slc26a4* p.T721M variant, demonstrated normal audiovestibular phenotypes and inner ear morphologies, similar to wild-type mice. To investigate whether the p.T721M variant could contribute to SNHI in mice through the haplo-insufficiency mode, we further generated mice with compound heterozygous variants (*Slc26a4*^*919-2A*>*G/T721M*^) by intercrossing *Slc26a4*^+*/T721M*^ mice with *Slc26a4*^*919-2A*>*G/919-2A*>*G*^ mice, which segregated the c.919-2A > G variant with abolished function. Compound heterozygous mice for p.T721M and c.919-2A > G (i.e., *Slc26a4*^*919-2A*>*G/T721M*^ mice) also had normal audiovestibular phenotypes, indicating that a single p.T721M allele was sufficient to maintain normal inner ear physiology in the mice.

The *SLC26A4* p.T721M variant has been documented sporadically in hearing-impaired families worldwide, including two Mediterranean families^18^, two Iranian families^19^, two Japanese family^20,21^, three Chinese families^22,23^, and one Taiwanese family^24^. Although this variant is widely distributed across different populations, its prevalence is relatively low compared to other *SLC26A4* variants, such as c.919-2A > G or p.H723R. According to the American College of Medical Genetics and Genomics (ACMG) guidelines in the Varsome platform^25^, *SLC26A4* p.T721M is classified as “pathogenic” by fulfilling the criteria of PM1, PM2, PP2, PP3, and PP5. It is located in the hotspot region of the *SLC26A4* gene, where the majority of pathogenic variants occur (PM1). Its allele frequency is < 0.0001 across various ethnic groups in gnomAD (PM2). The majority of non-VUS missense variants in *SLC26A4* have been reported as “pathogenic” in UniProt (PP2). In well-established databases (e.g., ClinVar, DVD^26^, and UniProt) and in several prediction algorithms (e.g., SIFT, Polyphen2, LRT, FATHMM, Mutation Taster, etc.), p.T721M was categorized as “pathogenic” (PP3 and PP5). Clinically, both homozygosity for p.T721M and compound heterozygosity for p.T721M with another *SLC26A4* mutation have been linked to non-syndromic DFNB4 or PS, as characterized by EVA, progressive or fluctuating severe-to-profound SNHI, and/or goiter^18–21^. Specifically, the hearing features have been detailed in two compound heterozygotes, one with symmetric SNHI (right/left: 103.75/110 dBHL)^21^, and the other with asymmetric SNHI (right/left: 112.5/68.75 dBHL)^20^. These typical clinical manifestations in p.T721M homozygotes and compound heterozygotes suggest that p.T721M is a pathogenic *SLC26A4* variant in humans.

In previous cell line studies, a number of *SLC26A4* variants, including p.P123S, p.M147V, p.L236P, p.S657N, p.T721M, and p.H723R, have been demonstrated to confer pathogenicity by affecting the trafficking process, rather than the expression level of pendrin^15,17^. However, the affected trafficking process can be rescued by certain treatments^15,17^. Low-temperature incubation has been reported to rescue the trafficking of pendrin with p.H723R, which originally accumulated in the endoplasmic reticulum, but not the trafficking of pendrin with p.L236P, which originally accumulated in centrosomal regions^17^. Similarly, salicylate treatment could rescue the trafficking of pendrin with p.P123S, p.M147V, p.S657N, and p.H723R, and restore the function of pendrin as an anion exchanger, but not that of pendrin with p.T721M^15^. These lines of evidence also support the view that the *SLC26A4* p.T721M variant is pathogenic and implies that p.T721M is more pathogenic compared to other missense *SLC26A4* variants, such as p.H723R.

To our surprise, the pathogenicity of p.T721M as predicted by the ACMG guidelines and evidenced by the cell line studies was not observed in our mouse model with the *Slc26a4* p.T721M variant. These findings are consistent with our previous studies in mouse models with *Slc26a4* p.H723R^10^ and p.C565Y^12^ variants. In the cochlea of mice, pendrin is expressed at the spiral prominence and outer sulcus cells^27^, which is almost the same as its expression in the cochlea of primates^28^. Therefore, the position of pendrin expression does not appear to be a major factor contributing to the phenotypic discrepancy between species.

Alternatively, the inter-species phenotypic discrepancy may be attributed to the structural differences between mouse and human pendrin.

**Figure 5:**
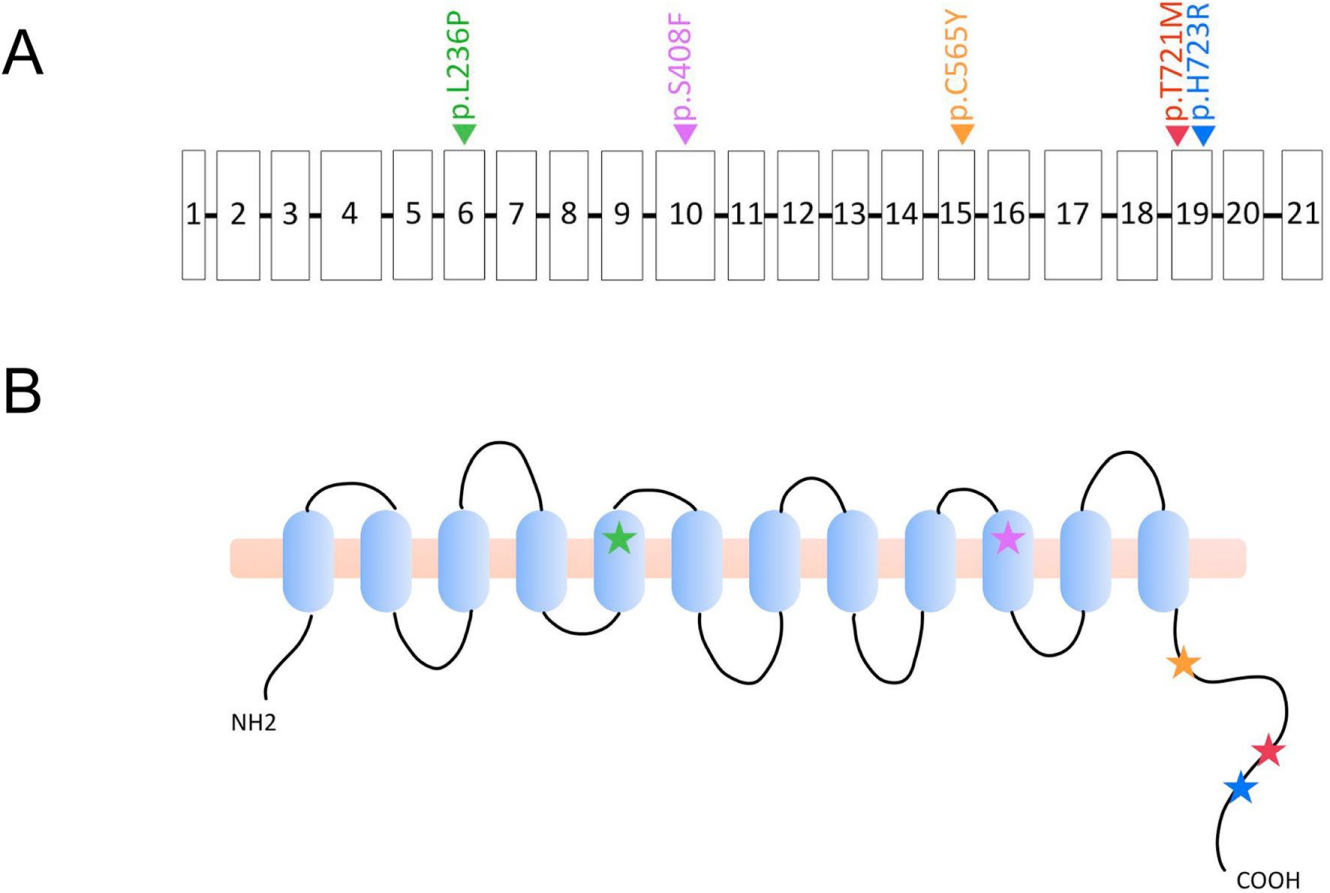
Five documented murine missense *Slc26a4* variants. Five mouse models with missense *Slc26a4* variants, including p.L236P, p.S408F, p.C565Y, p.T721M, and p.H723R, have been documented in the literature. (**A**) Scheme of the genomic DNA. Squares represent exons and lines represent introns. (**B**) Scheme of the 12-transmembrane domain model of pendrin. The five missense variants are indicated with stars, with p.L236P indicated in green, p.S408F in purple, p.C565Y in orange, p.T721M in red, and p.H723R in blue. L236P and p.S408F are located in the transmembrane domains, whereas p.C565Y, p.T721M, and p.H723R are located at the C-terminus of pendrin.

To date, five mouse models with missense *Slc26a4* variants, including p.L236P^11^, p.S408F^8^, p.C565Y^12^, p.T721M (this study), and p.H723R^10^, have been documented in the literature (Fig. 5A). Abnormal audiovestibular phenotypes were observed in mice with p.L236P and p.S408F, but not in mice with p.C565Y, p.T721M, and p.H723R. Notably, p.L236P and p.S408F are located in the transmembrane domains of pendrin^27,28^. In contrast, p.C565Y, p.T721M, and p.H723R are located in the C-terminus of pendrin comprised of amino acid residues 508–780^27,28^ (Fig. 5B). From an evolutionary perspective, the amino acid sequence of the pendrin C-terminus is less conserved, sharing only 86% identity between mice and humans. In contrast, the amino acid sequence of the transmembrane domains shared 92% identity between the two species (https://www.expasy.org/&https://www.uniprot.org/). Accordingly, we speculate that the absence of phenotypes in mice with *Slc26a4* p.C565Y, p.T721M, and p.H723R variants could be attributed to different protein structures at the C-terminus of human and mouse pendrin.

Another line of evidence that supports our speculation is the hH723R Tg mouse model^14^. Instead of creating the variant on murine genomic DNA, the authors delivered a sequence of human cDNA harboring the p.H723R variant into pronuclear-stage mouse embryos. This humanized transgenic mouse model revealed profound SNHI^10^, suggesting that C-terminus *SLC26A4* variants identified in humans might require a human peptide backbone to exhibit their pathogenicity.

Rapp et al. analyzed the locations of *SLC26A4* mutations and identified a high density of *SLC26A4* mutations in the anion-binding transmembrane domains (TMs), including TM1, 3, 8, and 10^29^. The findings indicate that when present in these transmembrane domains, *SLC26A4* variants are more likely to be pathogenic. This could possibly explain the higher evolutionary conservation of amino acid sequences between human and mouse pendrin at the transmembrane domains, as well as the clear phenotypic manifestation of profound SNHI in mice with *Slc26a4* variants at the transmembrane domains, such as *Slc26a4*^*L236P/L236P*^ and *Slc26a4*^*loop/loop*^ mice (i.e., mice with p.S408F).

In summary, using a genotype-driven approach, we generated a knock-in mouse model segregating the deafness-associated *SLC26A4* p.T721M variant in humans. Surprisingly, mice with the *Slc26a4* p.T721M variant exhibited a normal audiovestibular phenotype and inner ear morphology. Because there might be differences in the pathogenicity of specific *SLC26A4* variants between humans and mice, caution is needed while extrapolating the results of animal studies to humans.

## Methods

### Ethics statement

All animal experiments were carried out in accordance with animal welfare guidelines and approved by the Institutional Animal Care and Use Committee of the National Taiwan University College of Medicine (approval no. 20160337). Also, all animal experiments followed the guidelines of ARRIVE 2.0.

### Construction of ***Slc26a4***^***T721M/T721M***^ knock-in mice

Transgenic mice were generated by the Transgenic Mouse Models Core (TMMC, Taiwan) using the CRISPR technology-associated RNA-guided endonuclease Cas9 to mutate the *Slc26a4* gene and generate the *Slc26a4*^+*/T721M*^ mouse line. Specific guiding RNAs (sgRNAs) were developed to target exon 15 of the *Slc26a4* gene in C57BL/6 mice. The sgRNA and CRISPR/Cas9 RNA were delivered into the mouse zygote to generate founders. The two male founder mice that were obtained from each, harbored the p.T721M (c.2162C > T) variant in the *Slc26a4* gene. After germline transmission of the targeted variant allele, we produced the congenic *Slc26a4*^+*/T721M*^ mouse line used in this study by repeated backcrossing into the C57BL/6 inbred strain for 6–10 generations. Mice homozygous for the variant (*Slc26a4*^*T721M/T721M*^) were obtained by intercrossing heterozygous mice (i.e., *Slc26a4*^+*/T721M*^) (Fig. 1). Reverse transcription-PCR (RT-PCR) of mRNA of inner ear extract and direct sequencing confirmed a pure non-chimeric genetic background without unintentional wild-type *Slc26a4* expression in *Slc26a4*^*T721M/T721M*^ mice.

### Auditory evaluations

Mice were anesthetized and placed in a head-holder within an acoustically and electrically insulated and grounded test room^10^. We used an evoked potential detection system (Smart EP 3.90; Intelligent Hearing Systems, Miami, FL, USA) to measure auditory brainstem response (ABR) thresholds in mice. Click sounds, as well as 8, 16, and 32 kHz tone bursts at various intensities were generated to evoke ABRs. Response signals were detected using subcutaneous needle electrodes. The active electrodes were inserted into the vertex and the ipsilateral retroauricular region with a ground electrode on the back of each mouse. The ABRs were recorded from postnatal 12–44 weeks, to trace changes in auditory function.

ABRs were measured bilaterally. As there were no significant differences between the two ears, only the data of the left ears were presented and analyzed.

### Inner ear morphology studies

For light microscopy studies, tissues were stained with hematoxylin and eosin (H&E). The morphology of each sample was examined using a Leica optical microscope^10^. First, inner ear tissues from adult mice (P28-P30) were fixed by perilymphatic perfusion with 4% paraformaldehyde (PFA) in phosphate-buffered saline (PBS) through round and oval windows, and a small fenestra in the apex of the cochlear bony capsule. The specimens were decalcified for one week. The samples were then dehydrated and embedded in paraffin. Subsequently, serial Sects. (7 mm) were stained with H&E. ImageJ software (NIH, http://imagej.nih.gov/ij/download.html) was used to quantify the cross-sectional area of the middle turn scala media.

Whole-mount studies of mouse inner ear specimens were performed as previously described^10,30^. The tissues were stained with rabbit anti-Myosin-VIIA primary antibody (1:200; Proteus Bioscience Inc., Ramona, CA, USA). Later, the tissue sections were incubated with 4',6-diamidino-2-phenylindole (DAPI; 1:5000; Thermo Fisher Scientific, Waltham, MA, USA) and Alexa Fluor 568-conjugated goat anti-rabbit IgG (H + L) secondary antibodies (1:200; Thermo Fisher Scientific) at 4 °C overnight. After washing in PBS, the tissues were mounted using the ProLong Antifade kit (Molecular Probes, Eugene, OR, USA) for 20 min at room temperature. Images of the tissues were obtained using a model LSM 800 laser scanning confocal microscope (Carl Zeiss, Oberkochen, Germany).

### Expression of pendrin

Tissue sections were prepared from the inner ears of wild-type, *Slc26a4*^*919-2A*>*G/919-2A*>*G*^, and *Slc26a4*^*T721M/T721M*^ mice. The sections were mounted on silane-coated glass slides, deparaffinized in xylene, and rehydrated in ethanol. Tissues were stained with a 1:1000 dilution of rabbit anti-pendrin antibody kindly provided by Dr. Jinsei Jung, Yonsei University College of Medicine, Seoul, Republic of Korea. This was followed by exposure to DAPI (1:5000) and Alexa Fluor 488-conjugated goat anti-rabbit IgG (H + L) secondary antibody (1:200; Thermo Fisher Scientific). After incubation, the slides were washed with PBS and mounted with ProLong Antifade kit at 25 °C. Images were obtained using the aforementioned LSM 880 laser scanning confocal microscope^10^.

### Vestibular function evaluation

Mice were subjected to a series of tests at 8 weeks, including the swimming and rotarod tests. For the swimming test, the swimming performance of the mice was scored from 0 to 3, with 0 representing normal swimming and 3 representing underwater tumbling^31^. For the rotarod tests, the mice were placed on the rotating rod for a maximum of 180 s. The speed of the rods was accelerated from 5 rpm to a maximum speed of 20 rpm in one min. The length of time each mouse remained on the rotating rod was recorded^32^.

### Statistical analyses

Data are presented as the mean ± standard deviation. Statistical analyses were conducted using an unpaired Student’s *t*-test with Bonferroni correction for continuous variables. Statistical significance was set at *p* < 0.05. All analyses were performed using SPSS software (version 15.0; SPSS Inc., Chicago, IL, USA).

## Data Availability

The authors confirm that the data supporting the findings of this study are available within the article.
